# Schema Therapy for Voice Hearers: A Case Series Study

**DOI:** 10.1002/cpp.70349

**Published:** 2026-10-07

**Authors:** Georgie Paulik, Caitlin Reddyhough, Christopher W. Lee

**Affiliations:** ^1^ School of Psychology Murdoch University Murdoch Western Australia Australia; ^2^ Perth Voices Clinic Murdoch Western Australia Australia; ^3^ School of Psychology Curtin University Perth Western Australia Australia; ^4^ School of Health and Medical Sciences University of Western Australia Australia

**Keywords:** auditory verbal hallucinations, case series, chair‐work, imagery rescripting, schema therapy, voice hearing

## Abstract

Distressing voice‐hearing experiences are frequently characterised by hostility, criticism and abuse and emerging evidence suggests that these experiences may reflect internalised dysfunctional schema modes, particularly the Punitive Parent mode. Yet no research has evaluated a schema therapy intervention explicitly targeting schema modes. This naturalistic case series evaluated schema therapy for voices (STv) within a routine specialist psychology service. Twenty transdiagnostic voice hearers started formulation‐driven STv comprising approximately 20–30 individual sessions integrating chair‐work, imagery rescripting and schema mode work. Fifteen participants completed treatment. Primary outcome was voice‐related distress. Secondary outcomes included voice severity, voice content, negative affect, emotion regulation difficulties and schema modes. Feasibility indicators were encouraging, with 83.3% of eligible clients selecting STv and 75.0% completing treatment. Participants demonstrated a significant and large reduction in voice‐related distress (*d* = 1.18). Significant improvements were also observed in negative affect, emotion regulation difficulties and Punitive Parent mode. Changes in voice severity, negative voice content and Vulnerable Child mode were in the expected direction but did not reach statistical significance, while Healthy Adult mode showed little change. STv appears feasible to deliver within routine clinical practice and associated with meaningful reductions in voice‐related distress. The pattern of findings provides preliminary support for a schema mode conceptualisation of distressing voices and suggests that directly targeting Punitive Parent, Vulnerable Child and Healthy Adult modes may represent a promising therapeutic approach. Controlled trials are now required to establish efficacy and investigate proposed mechanisms of change.

## Summary

- Distressing voices may be usefully formulated within a schema mode framework, particularly as manifestations of Punitive Parent modes.
- Schema therapy adapted for voice hearers was feasible to deliver within a routine specialist psychological service using a focused 20–30 session intervention.
- Large reductions in voice‐related distress were observed despite more modest changes in voice severity, suggesting that targeting the punitive nature of the dominant voice and the way people relate to their voices may be important therapeutic targets.
- Chair‐work, imagery rescripting and Healthy Adult strengthening offer practical experiential methods for targeting punitive voices and unmet emotional needs.
- Although findings are preliminary, schema therapy for voices represents a promising mechanism‐informed approach that warrants evaluation in controlled trials.

## 1 Introduction

‘You stupid, stupid little girl’, says Martha, Tilly's dominant voice. I ask Tilly whether she notices any thoughts, images or memories while Martha says these hurtful words. Tilly replies, ‘When I hear her say that it almost transports me back to being a preschooler, my mum shaking her head at me, saying those same words because I'd done something wrong, like spilling my breakfast on my new vest. I can remember the look she would give me—utter disgust and disappointment. I know I'm pathetic. And the voice I hear now, Martha, reminds me of that every day’.

Tilly's experience of hearing a hostile, critical and punitive dominant voice is common among help‐seeking voice hearers (e.g., Bartulovich et al. 2025). Likewise, histories of childhood adversity are highly prevalent in transdiagnostic voice hearing populations and are frequently associated with the development and maintenance of distressing voices (Strachan et al. 2022; Strachan, Paulik, and McEvoy 2024). These associations may be direct, whereby voices repeat the exact words spoken by perpetrators of abuse, or, as in Tilly's case, indirect, whereby voice content reflects the person's internalised beliefs or schemas—broad, enduring patterns of meaning about the self, others and relationships that develop through experience—following adverse childhood experiences (Hardy et al. 2005). Rather than representing random perceptual experiences, distressing voices often appear to communicate longstanding interpersonal themes of criticism, rejection, shame, threat and worthlessness. Although voice hearing can be a distressing experience, it is not inherently pathological or in need of treatment; psychological intervention is typically sought when voices are distressing, disruptive or interfere with valued functioning. Accordingly, the present intervention targets voice‐related distress and the person's relationship with their voices rather than voice hearing per se.

These observations have increasingly shifted psychological models of voice hearing towards understanding voices as meaningful psychological experiences rooted in autobiographical memory, attachment and trauma (Longden, Corstens, et al. 2012; Longden, Madill, and Waterman 2012; Strachan, Paulik, and McEvoy 2024). One therapeutic implication is that directly modifying trauma memories and their associated meanings or schemas—such as the memories Tilly identified of her mother—may alter the emotional and interpersonal processes that maintain distressing voices. Building on this rationale, Paulik and colleagues (2019; Strachan, McEvoy, Arntz, et al. 2024) investigated the use of Imagery Rescripting (ImRs) for trauma‐affected voice hearers. ImRs is a trauma‐focused, experiential cognitive therapy in which distressing autobiographical memories are deliberately reactivated and imaginatively modified so that unmet emotional needs are addressed, new meanings are generated, and corrective emotional experiences are encoded (Arntz 2025). Across the initial case series (Paulik et al. 2019) and subsequent open trial (Strachan, McEvoy, Arntz, et al. 2024), participants demonstrated large reductions in voice‐related distress and severity, alongside substantial reductions in trauma intrusions that were maintained at three‐month follow‐up. Although preliminary and requiring replication in larger controlled trials, these findings are particularly encouraging given that cognitive behavioural therapy (CBT) for distressing voices—the current gold‐standard psychological intervention—typically demonstrates only modest effects (Hayward et al. 2017; Thomas et al. 2011).

Interestingly, qualitative interviews conducted following the open trial suggested that participants perceived the benefits of ImRs to extend beyond changes in traumatic memories alone. Participants described feeling safer, more confident in their ability to cope, less dominated by their voices and increasingly able to challenge and distance themselves from hostile voice content (Strachan et al. 2023). These findings raise an important clinical question. If directly modifying traumatic memories can reduce the emotional impact of distressing voices, might outcomes be further enhanced by embedding ImRs within a broader therapeutic approach?

Schema therapy provides one such framework that also explicitly targets the internalised critical relationship represented by many voices, actively strengthens an individual's sense of internal safety and self‐compassion, and directly challenges the dominance of hostile voices through additional experiential techniques such as chair‐work. Schema therapy is an integrative psychotherapy originally developed for chronic psychological difficulties and personality disorders (Young et al. 2003). It combines cognitive and behavioural methods with attachment, developmental and emotion‐focused approaches, such as ImRs and chair‐work. Central to Schema therapy are early maladaptive schemas and schema modes. Early maladaptive schemas are enduring patterns of memories, emotions, cognitions and bodily sensations concerning the self and relationships with others that develop when core emotional needs are not adequately met. Schema modes are the moment‐to‐moment emotional, cognitive, behavioural and physiological states that arise when one or more schemas and associated coping responses are activated. Across qualitative, quantitative and clinical/theoretical work, it has been proposed that voices may not only activate schema modes within the hearer—such as maladaptive coping modes—but may themselves represent or embody specific schema modes (Baelemans et al. 2025; Bartulovich et al. 2025; Newton et al. 2026; Whitehead 2018).

Compared with nonclinical and Axis I reference groups, voice hearers were found to have higher levels of pathology on three modes: Punitive Parent, Healthy Adult and Vulnerable Child (Newton et al. 2026). The Punitive Parent mode represents internalised harsh, critical, shaming or abusive messages, often originating in adverse early relationships (Young et al. 2003), and has also been specifically linked with voice‐hearing experiences in people receiving schema therapy for BPD (Baelemans et al. 2025). It attacks or punishes the person for perceived mistakes, needs, emotions or personal characteristics and may promote self‐hatred, shame, self‐punishment and self‐harm. The Vulnerable Child mode reflects states of fear, sadness, helplessness, shame, loneliness and unmet attachment needs. In contrast, the Healthy Adult mode supports balanced thinking, emotional regulation, adaptive problem‐solving, self‐compassion and appropriate boundary setting; it also protects and nurtures the Vulnerable Child and limits the influence of dysfunctional modes.

Furthermore, qualitative work has identified that voice content can map onto Punitive Parent, Vulnerable Child and Healthy Adult modes (Bartulovich et al. 2025). Other researchers and clinicians have also investigated applying the schema mode framework to voice hearing (Baelemans et al. 2025; Newton et al. 2026; Rhodes and Vorontsova 2024; Salt et al. 2024; Whitehead 2018). Consistent with this schema‐mode account, negative voices commonly criticise, blame, humiliate, threaten or punish the hearer (Berry et al. 2012; Moskowitz et al. 2017; Nayani and David 1996) and may resemble abusive caregivers, bullies or perpetrators from the person's past (Corstens and Longden 2013; Peach et al. 2021; Van Den Berg et al. 2023). These characteristics closely parallel the Punitive Parent mode, which is itself conceptualised as an internalised critical or punitive ‘voice’ (Young et al. 2003). Voices may also exhibit features consistent with other schema modes. For example, voices characterised by fear, sadness, pleading, helplessness or fear of rejection may resemble the Vulnerable Child mode (Daalman et al. 2012; Diederen and Sommer 2012; Nayani and David 1996; Van Den Berg et al. 2023), while supportive, reassuring, protective or encouraging voices may share characteristics with the Healthy Adult mode (Jenner et al. 2008; Salt et al. 2024).

Related evidence has emerged from research with people diagnosed with borderline personality disorder (BPD). In a qualitative study of 16 people who had received schema therapy, Baelemans et al. (2025) found that approximately half described auditory verbal hallucinations as manifestations of the Punitive Parent mode before treatment. Participants characterised this mode as involving pervasive self‐critical and punitive messages that contributed to emotional distress and maladaptive coping. They reported that schema therapy techniques, particularly ImRs and chair‐work, reduced the power and credibility of the Punitive Parent mode and associated voices. Although this study was retrospective and restricted to people with BPD, it provides preliminary evidence that some voices may be experienced as externalised or perceptually vivid expressions of internalised punitive modes and that directly targeting these modes may alter the voice hearing experience.

Taken together, this emerging literature suggests a clinically meaningful interaction between punitive voices, vulnerable emotional states and limited access to adaptive self‐regulation. From a schema therapy perspective, distressing voice hearing may be maintained by a dynamic in which a Punitive Parent‐like voice attacks or threatens the person, activates the Vulnerable Child mode, and overwhelms an insufficiently developed or accessible Healthy Adult mode. This formulation provides a rationale for an intervention that directly limits and weakens punitive modes and associated voices, attends to the unmet needs and emotional pain of the Vulnerable Child, and strengthens the Healthy Adult's capacity to provide protection, validation, regulation and adaptive guidance.

### 1.1 Aims and Hypotheses

The present study aimed to conduct a preliminary evaluation of schema therapy for voices (STv) when delivered to transdiagnostic voice hearers within a routine clinical service. The first aim was to evaluate the feasibility of delivering STv to help‐seeking voice hearers within routine clinical practice; no inferential hypothesis was specified for this descriptive feasibility aim. The second aim was to evaluate preliminary change in the primary outcome of voice‐related distress, with the hypothesis that distress would decrease from pretreatment to posttreatment. The third aim was to examine broader secondary clinical outcomes (voice severity, positive and negative dominant‐voice content, negative affect and emotion regulation difficulties); given the preliminary nature of the study, these analyses were exploratory. Finally, analyses of schema modes were restricted a priori to Punitive Parent, Vulnerable Child and Healthy Adult modes because these were the modes most directly targeted by STv and implicated in emerging voice‐hearing research. We hypothesised improvement across these three mode scores over treatment.

## 2 Method

### 2.1 Design

The study employed a naturalistic, uncontrolled case series design and was conducted as a routine service evaluation within an outpatient specialist psychology service for people experiencing distressing voices and other hallucinations. STv was delivered individually over approximately 20–30 sessions, with treatment length varying according to formulation and clinical need. Outcomes were assessed at baseline, midtreatment (Session 10) and posttreatment.

### 2.2 Participants

Participants were 20 help‐seeking voice hearers who commenced STv at the Perth Voices Clinic. Inclusion criteria were: (1) currently experiencing distressing voices; and (2) access to sufficient funding to attend 20–30 therapy sessions. Eligibility was not restricted according to psychiatric diagnosis; however, because STv was offered specifically to voice hearers with substantial emotion‐regulation difficulties and clinically meaningful links between childhood adversity and current voices, the resulting sample should be understood as diagnostically heterogeneous but clinically selected, rather than representative of an unselected transdiagnostic voice‐hearing population. Exclusion criteria were: (1) experiencing an acute phase of psychosis (nb. voice hearing alone did not constitute an acute phase of psychosis; exclusion applied when clinical assessment indicated acute psychotic deterioration of sufficient severity to require stabilisation or a higher level of care before commencing psychological therapy); (2) high acute risk of harm to self or others; and (3) marked intellectual impairment (defined clinically as impairment of a degree that would preclude meaningful engagement with the assessment, formulation and individual psychotherapy; a formal IQ cut‐off was not applied). Consistent with the transdiagnostic nature of the service, eligibility was not restricted according to psychiatric diagnosis.

Participants were help‐seeking voice hearers attending the specialist service and were offered STv following the standard clinical assessment when the intervention was judged clinically appropriate; treatment selection was then made collaboratively according to clinical formulation and client preference (see Procedure). Of the 20 participants who commenced STv, 15 completed treatment. Demographic and clinical characteristics of treatment completers and noncompleters are presented in Table 1.

**TABLE 1:** Demographics and clinical characteristics of completers and noncompleters.

	Completers (*N* = 15)		Noncompleters (*N* = 5)	
Characteristics	Count, M (SD)	%	Count, M (SD)	%
Mean HPSVQ total score	21.33 (5.86)	—	17.25 (8.77)	—
Mean duration of voice hearing in years (SD) (range)	19.33 (13.36) (7–56)	—	11 (6.04) (5–20)	—
Mean age in years (SD)	35.93 (13.06)	—	31.8 (8.64)	—
**Gender**				
Female	6	40%	4	80%
Male	5	33.33%	1	20%
Nonbinary	3	20%		
Prefer to self‐describe	1	6.67%	0	0%
**Marital status**				
Married/cohabiting/long term relationship	6	40%	2	40%
Without a partner	9	60%	3	60%
**Employment**				
Total unemployed	4	26.67%	3	60%
Full time/part time paid employment	8	53.33%	0	0%
Other (includes student)	3	20%	2	40%
**Education**				
Left school 16 years (year 11)	1	6.67%	3	60%
Left school 17/18 years (year 12)	11	73.33%	2	40%
Completed college	2	13.33%	0	0%
Completed university/postgrad	1	6.67%	0	0%
**Ethnicity**				
White	12	80%	5	100%
Aboriginal or Torres Strait Islander	2	13.33%	0	0%
Other	1	6.67%	0	0%
**Diagnosis** ^**a**^				
Psychotic disorder only	3	20%	1	20%
Psychotic disorder with co‐morbid diagnoses^b^ (not BPD)	2	13.33%	1	20%
Psychotic disorder + BPD	1	6.67%	2	40%
BPD only	0	0%	0	0%
BPD with co‐morbid diagnoses other than psychosis	6	40%	0	0
Nonpsychotic or BPD diagnoses^a^	2	13.33%	0	0%
No diagnosis/Not provided	1	6.67%	1	20%
**Psychotropic medication**				
Yes	15	100%	5	100%
No medication	0	0%	0	0%

*Note:* HPSVQ = Hamilton Program for Schizophrenia Voices Questionnaire; BPD = Borderline Personality Disorder (also including Emotionally Unstable Personality Disorder).

^a^ Diagnostic categories were based on diagnoses reported on referral; psychosis subtype and nonvoice psychotic symptoms were not systematically coded for the service evaluation.

^b^ Single or multiple diagnoses.

### 2.3 Measures

Self‐report measures were completed at baseline, midtreatment (Session 10) and posttreatment. Baseline measures were completed following the initial assessment session, posttreatment measures were completed prior to the final therapy session to enable participants and therapists to review and discuss changes over the course of treatment. Participants completed measures online using Qualtrics or, according to participant preference, using paper‐and‐pencil questionnaires. Given the number of routinely collected measures and the small sample, analyses were restricted to prespecified clinically and theoretically relevant outcomes to limit the number of statistical comparisons and associated Type I error risk.

#### 2.3.1 Primary Outcome Measure

##### 2.3.1.1 Voice‐Related Distress

The Hamilton Program for Schizophrenia Voices Questionnaire (HPSVQ; Van Lieshout and Goldberg 2007) is a nine‐item self‐report measure assessing the severity and emotional impact of voice hearing experiences. Items are rated on a 5‐point scale ranging from 0 (*least severe or impairing*) to 4 (*most severe or impairing*). The four‐item Emotional Characteristics of Voices subscale was used as the primary outcome measure of voice‐related distress, consistent with previous research (Berry et al. 2021). Scores range from 0 to 16, with higher scores indicating greater voice‐related distress. The HPSVQ has demonstrated good reliability and validity in voice hearing populations (Berry et al. 2021; Kim et al. 2010; Ratcliff et al. 2011).

#### 2.3.2 Secondary Outcome Measures

##### 2.3.2.1 Voice Severity

The five‐item Physical Characteristics of Voices subscale of the HPSVQ (Van Lieshout and Goldberg 2007) was used to assess voice severity, including frequency and duration of voices, loudness, clarity and the extent to which voices interfere with or intrude on experience. Scores range from 0 to 20, with higher scores indicating greater voice severity.

##### 2.3.2.2 Voice Content

The Perth Voice Content Questionnaire (PVCQ; Salt et al. 2024) is a 20‐item self‐report measure assessing the positive and negative content of participants' dominant voice. Items are rated on a 5‐point scale ranging from 0 (*not at all*) to 4 (*extremely*). Separate Positive and Negative Voice Content subscale scores range from 0 to 40. Higher scores indicate greater positive or negative voice content, respectively. Preliminary psychometric evaluation has supported the reliability and validity of the PVCQ (Salt et al. 2024).

##### 2.3.2.3 Negative Affect

The Depression Anxiety and Stress Scales–21 (DASS‐21; Lovibond and Lovibond 1995) is a 21‐item self‐report measure assessing symptoms of depression, anxiety and stress. Items are rated on a 4‐point scale ranging from 0 (*did not apply to me at all*) to 3 (*applied to me very much, or most of the time*). Only the total score was reported, with higher scores indicating greater symptom severity.

##### 2.3.2.4 Emotion Regulation Difficulties

The Difficulties in Emotion Regulation Scale–Short Form (DERS‐SF; Kaufman et al. 2016) is an 18‐item self‐report measure assessing difficulties with emotion regulation. Items are rated on a 5‐point scale ranging from 1 (*almost never*) to 5 (*almost always*), yielding possible scores ranging from 18 to 90. Higher scores indicate greater difficulties with emotion regulation.

##### 2.3.2.5 Schema Modes

The Schema Mode Inventory (SMI Version 1.1; Young et al. 2009) is a 124‐item self‐report measure assessing 14 schema modes. Items are rated on a 6‐point scale ranging from 1 (never or almost never) to 6 (all of the time). The SMI has demonstrated good internal consistency and construct validity (Lobbestael et al. 2010). Although the complete SMI was administered to inform participants' clinical formulations, analyses were restricted a priori to the three modes most directly targeted by STv: Punitive Parent, Vulnerable Child and Healthy Adult modes. Scores for each mode were calculated by averaging the relevant items, with possible scores ranging from 1 to 6. Higher scores indicate greater endorsement of the respective schema mode.

### 2.4 Intervention

STv was developed by the study team as a formulation‐driven adaptation of standard schema therapy for help‐seeking voice hearers, drawing on established schema therapy principles and experiential methods (Young et al. 2003), our previous work using ImRs with trauma‐affected voice hearers, and emerging work linking voice content with schema modes. The adaptation retained core schema therapy methods while explicitly incorporating voices into mode formulation and experiential work.

STv was delivered individually over 20–30 weekly 60‐min sessions. STv is a formulation‐driven intervention adapted for people experiencing distressing voices and associated emotion regulation difficulties. The primary aims of the intervention are to identify links between participants' voices, early maladaptive schemas and schema modes, as well as to directly target the dysfunctional parent modes (primarily Punitive Parent mode) and associated voices that are hypothesised to contribute to voice‐related distress. Treatment also aimed to strengthen the Healthy Adult mode, increase awareness of and responsiveness to the needs underlying Vulnerable Child mode and modify maladaptive coping modes where these interfere with therapeutic change. Voices are incorporated directly into the schema mode formulation and, where appropriate, conceptualised as representing or amplifying particular modes. Key experiential techniques include chair‐work, used to explore and modify relationships between modes and associated voices, and ImRs, used to target memories linked to the development and maintenance of dysfunctional modes and voices. The broad structure of the intervention is summarised in Table 2. Further discussion of the STv intervention structure is outlined in the Supplementary Material.

**TABLE 2:** Overview of STv sessions.

Approximate sessions	Treatment focus
Assessment (Sessions 1–2)	Assessment of voice hearing experiences, mental health and developmental history, trauma and attachment experiences, unmet emotional needs, early maladaptive schemas and initial treatment goals.
Sessions 2–6	Psychoeducation and exploration of schemas and modes; identification of links between developmental experiences, unmet needs, schemas, modes and current difficulties; exploration of voices and their relationship to schema modes; development of a collaborative mode map and individualised treatment goals.
Sessions 7–15	Primary intervention phase targeting dysfunctional parent/inner critic modes and associated voices, predominantly using chair‐work and ImRs; concurrent strengthening of the Healthy Adult mode and recognition of—and tending to—Vulnerable Child needs.
Sessions 15–18	Identification and modification of relevant maladaptive coping modes where these maintain difficulties or interfere with therapeutic work; further work addressing Vulnerable Child needs and strengthening the Healthy Adult mode.
Sessions 19–30, as indicated	Continued formulation‐driven work targeting clinically relevant modes and voices; consolidation of the Healthy Adult mode; development and rehearsal of strategies for responding to dysfunctional modes and voices; schema relapse prevention and treatment review.

### 2.5 Procedure

Participants were help‐seeking voice hearers referred to the Perth Voices Clinic (PVC), a specialist psychological service for people experiencing distressing voices and other hallucinations. All clients attending PVC completed a standardised initial clinical assessment designed to inform treatment planning and allocation to one of the psychological therapy streams offered by the service. The assessment included exploration of current and past voice hearing experiences and other mental health difficulties, developmental and trauma history, early attachment experiences and potential relationships between childhood adversity and the development or maintenance of voices.

The primary treatment stream offered at PVC was Imagery‐Enhanced Cognitive Behaviour Therapy for voices (IE‐CBTv). However, STv was considered as an alternative treatment option for clients who (1) had a diagnosis of BPD and/or experienced significant difficulties with emotion regulation that they identified as a treatment goal; (2) demonstrated a clinically meaningful relationship between childhood adversity and their current voice hearing experiences; and (3) had access to sufficient funding to attend approximately 20–30 therapy sessions. At the conclusion of the initial assessment, clients meeting these criteria were provided with information about the relevant treatment options available at PVC. Treatment selection was made collaboratively between the client and assessing clinician, with clients proceeding to STv where this was identified as a clinically appropriate option and was their preferred treatment approach.

Clients who commenced STv were offered 20–30 weekly, 60‐min individual therapy sessions. Outcome measures were administered at baseline, midtreatment (Session 10) and following completion of therapy. As the study was conducted as a service evaluation of routinely delivered clinical care, treatment duration and content were tailored to participants' individual clinical formulations and therapeutic needs within the framework of the STv treatment protocol.

There were two clinical psychologists delivering the therapy, both with advanced schema therapy training (one schema accredited). Clinical supervision was given by the last author (who is an accredited schema supervisor and trainer) once a fortnightly basis for the first 6 months of the study and then on a monthly basis until study completion.

### 2.6 Statistical Analysis

All statistical analyses were conducted using Jamovi (Version 2.6.44; The Jamovi Project 2024). A series of repeated‐measures analyses of variance (ANOVA) were conducted to examine change across baseline, midtreatment and posttreatment assessment points on the primary outcome measure (Voice Distress) and secondary outcomes (Voice Severity, Voice Content, Emotion Regulation Difficulties and Negative Affect).

To assess the clinical meaningfulness of change, reliable change indices were calculated for the primary outcome measure and the emotion regulation measure. Post‐estimation comparisons were conducted to evaluate differences between baseline and both midtreatment and posttreatment scores, with effect sizes quantified using Cohen's *d*.

For each repeated‐measures ANOVA, sphericity was assessed using Mauchly's test. When the assumption of sphericity was violated, adjusted degrees of freedom were applied using the Greenhouse–Geisser or Huynh–Feldt correction, as appropriate. Change in schema modes was examined using repeated‐measures ANOVA on the three schema‐related measures. All analyses were conducted on participants who completed the full course of treatment. For the variables with two time points (pretreatment and posttreatment), paired‐samples *t* tests were used.

## 3 Results

Demographic and clinical characteristics of treatment completers (*N* = 15) and noncompleters (*N* = 5) are presented in Table 1. Formal statistical comparisons were not conducted because of the small number of noncompleters. No clear patterns of demographic or clinical differences were evident, and any apparent differences should be interpreted cautiously given the small sample size of noncompleters.

### 3.1 Feasibility

During July 2022 to January 2025, 24 help‐seeking clients attending the specialist service met the clinical eligibility criteria and were offered STv; 20 selected STv and started treatment. Of the 24 clients who met eligibility criteria and were offered STv, 20 (83.3%) selected STv in preference to the Imagery‐Enhanced CBT or ImRs treatment streams. Of these, 15 (75.0%) completed the intervention. Of the five noncompleters, one changed treatment streams prior to completing baseline assessment measures and two changed treatment streams approximately five sessions into treatment, prior to commencing the primary experiential intervention phase. One participant withdrew following a deterioration in mental health, and one withdrew prior to their final treatment session following a rupture in the therapeutic relationship.

All 15 treatment completers completed outcome assessments at baseline, midtreatment and posttreatment, although there were small amounts of item‐ or measure‐level missing data across assessment points.

No clinically significant deterioration in the primary outcome was observed among treatment completers. Fourteen of the 15 completers demonstrated reductions in voice‐related distress, while the remaining participant showed only a one‐point increase on the HPSVQ Voice‐Related Distress subscale (10–11), which was not considered clinically meaningful. One completer engaged in an incident of nonsuicidal self‐injury (NSSI) early in treatment; following clinical review, the distress precipitating this incident was judged to be unrelated to the intervention. Among noncompleters, one participant engaged in NSSI and one experienced an exacerbation of psychotic symptoms (not captured in measures completed, but reported by client and observed by the treating clinician) requiring psychiatric hospitalisation, both of which were judged to be unrelated to the intervention. The deterioration in psychotic symptoms was considered most likely associated with changes in psychiatric medication. One further noncompleter engaged in NSSI that was considered potentially treatment‐related. This participant reported increased anxiety and suicidal ideation associated with the anticipated processing of traumatic experiences within STv and used NSSI to regulate the resulting distress. Overall, one adverse event was considered potentially related to the intervention. Clinically significant deterioration was examined separately as an individual‐level safety/feasibility indicator; the analyses below evaluate group‐level change in the primary outcome over time.

### 3.2 Primary Outcome

The repeated measure ANOVA indicated a significant effect of time on voice distress, (*F*(2, 28) = 10.2, *p* < 0.001, *η*_p_^2^ = 0.422, Cohen's *d* = 1.18). Estimated marginal means showed a reduction over time (see Figure 1). Post hoc comparisons (Bonferroni‐adjusted) indicated a significant decrease from pre to post *t* = 4.63, (*p* < 0.001) and from mid to post *t* = 3.46, (*p* < 0.01), with no significant difference between pre and mid (*t* = 1.05). These results suggest a robust reduction in distress associated with voices over the course of treatment.

**FIGURE 1:**
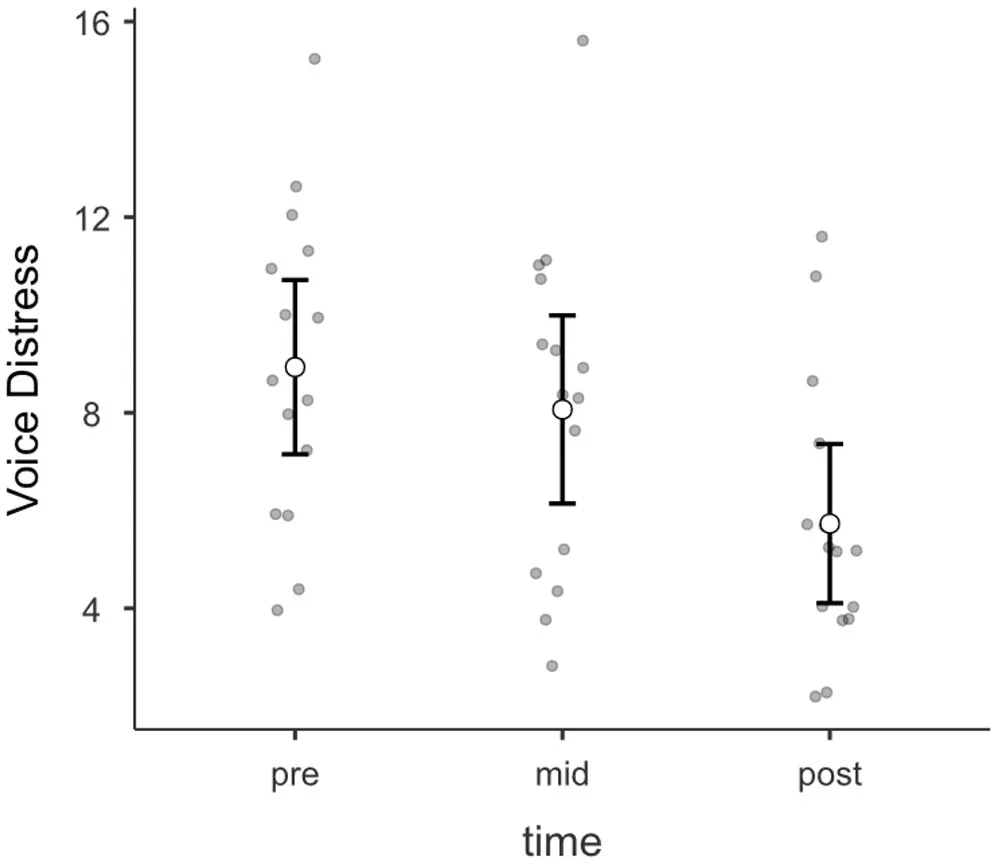
Scatter plot of voice distress scores showing mean value with 95 per cent confidence interval over time.

In assessing clinical reliable change pre to post, using a test–retest value of 0.72 (Van Lieshout and Goldberg 2007) and a reliable change threshold = ±1.96, improvement in voice distress scores reached the reliable change threshold for 10 participants, with no reliable change for five participants.

### 3.3 Secondary Outcomes

A repeated measures ANOVA revealed a significant effect of time on voice severity, *F*(2, 28) = 3.54, *p* = 0.043, *η*_p_^2^ = 0.202. However, Bonferroni‐corrected post hoc comparisons did not detect significant differences between any pair of time points (pre vs. mid, pre vs. post or mid vs. post; see Table 3). Although the omnibus test was significant and the effect size (*η*_p_^2^ = 0.20) met Cohen's (1988) threshold for a ‘large’ effect, the failure of the corrected pairwise comparisons to reach significance indicates that the treatment produced only a modest—rather than robust—effect on voice severity.

**TABLE 3:** Means (M) and standard errors (SE) for each secondary measure across time with *t* tests (and Cohen's *d*) for significant differences between time points.

Measure	Baseline M (SE)	Midtreatment M (SE)	End of treatment M (SE)	Pre vs. mid *T* value (Cohen's *d*)	Mid vs. post *T* value (Cohen's *d*)	Pre vs. post *T* value (Cohen's *d*)
Voice Severity	12.1 (0.91)	12.1 (0.73)	10.1 (0.98)	−0.00 (≈0.00)	2.25 (0.65)	2.08 (0.60)
Negative Voice Content^a^	19.4 (2.75)	17.4 (3.62)	11.9 (3.73)	0.94 (0.33)	1.52 (0.54)	*2.75 (0.97)*
Positive Voice Content^a^	7.87 (3.07)	11.50 (3.62)	6.12 (3.22)	−1.49 (−0.53)	2.52 (0.89)	0.47 (0.17)
DASS‐21 Total	72.7 (3.92)	61.3 (4.67)	45.5 (3.49)	1.87 (0.54)	4.73 (1.37) **	4.86 (1.41) **
DERS^a^	59.60 (3.97)	53.80 (4.33)	44.20 (3.56)	1.62 (0.49)	2.03 (0.62)	*3.03 (0.91)* *

*Note:* Voice Severity measured by the severity subscale of the Hamilton Program for Schizophrenia Voices Questionnaire; voice content measured by the Perth Voice Content Questionnaire.

Abbreviations: DASS = Depression Anxiety and Stress Scale; DERS = Difficulties in Emotion Regulation Scale–Short Form.

* *p* < 0.05.

** *p* < 0.01.

^a^ The DERS (*n* = 11) and the PVCQ for Negative and Positive Voice Content (*n* = 8) were introduced as measures several months into data collection, and thus not all 15 participants had baseline data collected on these measures. For all other measures, *n* = 15.

For negative voice content, the effect of time approached significance (*F*(2, 14) = 3.60, *p* = 0.055, *η*_p_^2^ = 0.340). Bonferroni‐corrected post hoc comparisons did not detect significant differences between any pair of time points (pre vs. mid, pre vs. post or mid vs. post; see Table 3). For positive voice content, the repeated‐measures ANOVA did not show a significant effect of time (*F*(2, 14) = 1.85, *p* = 0.193, *η*_p_^2^ = 0.209). Bonferroni‐corrected post hoc comparisons did not detect significant differences between any pair of time points (pre vs. mid, pre vs. post or mid vs. post; see Table 3).

A repeated‐measures ANOVA showed a significant effect of time on DASS total scores, (*F*(2, 28) = 14.0, *p* < 0.001, *η*_p_^2^ = 0.583). Post hoc comparisons (Bonferroni‐adjusted) indicated a significant decrease from pre to post, and from mid to post but not pre to mid (see Table 3.) Reliable Change Index (RCI) values were calculated for the DASS‐21 Total score using Cronbach's *α* = 0.93 (Henry and Crawford 2005). The standard error of measurement (SEM) was 3.97, and the standard error of the difference (SEdiff) was 5.62, yielding a reliable change threshold of ±11.02. Seven participants demonstrated reliable improvement from pretreatment to posttreatment, and no participants showed reliable deterioration. The remaining eight participants exhibited no reliable change.

A repeated‐measures ANOVA showed a significant effect of time on DERS scores (*F*(2, 18) = 5.96, *p* < 0.01, *η*_p_^2^ = 0.399). The post hoc comparisons (Bonferroni‐adjusted) are presented in Table 3 and they indicated a significant decrease from pre to post, but not from pre to mid or mid to post.

Pretreatment to posttreatment changes in the schema modes most directly targeted by STv are presented in Table 4. In accordance with the hypotheses, participants demonstrated a significant reduction in Punitive Parent mode scores from pretreatment to posttreatment, *t*(14) = 2.31, *p* = 0.037. Although Vulnerable Child mode scores also decreased over the course of treatment, this change did not reach statistical significance, *t*(14) = 1.78, *p* = 0.095. No significant change was observed in Healthy Adult mode scores, *t*(14) = 0.41, *p* = 0.68.

**TABLE 4:** Pre–post change on schema mode measures, including means, standard error of the difference (SE(diff.)), *t* scores, and *p* vales (* significant < 0.05).

Mode	Pre mean	Post mean	Mean difference	SE (diff.)	*t*(14)	*p*
Vulnerable Child	3.78	3.46	0.32	0.18	1.78	0.095
Punitive Parent	3.66	3.33	0.33	0.14	2.31	0.037*
Healthy Adult	3.77	3.72	0.05	0.12	0.41	0.68

*Note:* Schema modes measured using the Schema Mode Inventory.

## 4 Discussion

The present naturalistic case series provides preliminary support for the feasibility and potential clinical utility of adapting schema therapy to directly target distressing and punitive voices in transdiagnostic voice hearers. STv was associated with high treatment uptake and generally good retention, alongside a significant and large reduction in the primary outcome of voice‐related distress. Improvements were also observed across several secondary outcomes, including negative affect, emotion regulation difficulties and Punitive Parent mode. These findings are preliminary and cannot establish treatment efficacy given the uncontrolled service‐evaluation design; nevertheless, they provide an initial indication that a mode‐focused intervention incorporating ImRs and chair‐work can be delivered within routine practice and warrants more rigorous evaluation.

The first aim was to evaluate the feasibility of delivering STv within routine clinical practice. Feasibility indicators were broadly encouraging. Of the clients offered STv, 83.3% selected it in preference to the other available therapy streams, suggesting that the intervention was acceptable as a treatment option to most eligible clients. Fifteen of the 20 clients who commenced STv completed treatment, corresponding to a completion rate of 75% and a noncompletion rate of 25%. This rate is comparable to the 23.5% dropout rate reported for schema therapy in a recent 18‐month randomised trial for BPD, despite the present sample comprising transdiagnostic voice hearers with complex clinical presentations (Assmann et al. 2024). It is somewhat higher than the pooled dropout rate of approximately 18.8% reported across trials of CBT for psychotic disorders, although CBT for psychosis studies vary considerably in treatment length, setting, eligibility criteria and definitions of dropout, limiting direct comparison (Bighelli et al. 2018). The nature and timing of noncompletion provide additional context. Three clients changed treatment streams either before baseline assessment or approximately five sessions into STv, before beginning the primary experiential intervention phase, following collaborative reconsideration of their treatment needs and goals. Only two participants—10% of those who commenced STv—withdrew after progressing further into treatment for reasons associated with mental health deterioration or the therapeutic process. Thus, although the overall completion rate should remain the principal indicator of retention, not all instances of noncompletion appeared to reflect rejection of, or inability to tolerate, the active components of STv.

Safety findings were generally reassuring but warrant cautious interpretation. One adverse event was judged to be potentially related to treatment: a participant reported increased anxiety and suicidal ideation associated with anticipating trauma processing and engaged in NSSI to regulate this distress (though this was a frequent affect regulation strategy for this client prior to commencing therapy). No clinically significant deterioration was identified among treatment completers, and the other recorded incidents of NSSI or psychiatric deterioration were judged clinically to be unrelated to STv. Together, these findings suggest that STv can be delivered in routine practice with generally satisfactory uptake, retention, assessment completion and safety; however, the potentially treatment‐related adverse event highlights the need for careful preparation, collaborative pacing, ongoing risk monitoring and explicit discussion of concerns about experiential and trauma‐focused work.

The second aim was to evaluate preliminary changes in the primary outcome of voice‐related distress. Participants demonstrated a significant and large reduction in voice‐related distress over the course of treatment (Cohen's *d* = 1.18). Although the uncontrolled design precludes conclusions regarding treatment efficacy, the magnitude of improvement is encouraging. Meta‐analytic reviews have generally found CBT for psychosis to produce small‐to‐moderate improvements in hallucinations and related positive symptoms (effect sizes for Hedges' *g* ≈ 0.32–0.40 at the end of treatment; Berendsen et al. 2024). More recently, a meta‐analysis of a larger array of psychological interventions specifically targeting auditory hallucinations similarly concluded that these interventions produce only small‐to‐moderate effects overall (Fässler et al. 2026). Although direct comparisons should be interpreted cautiously because of study design differences, outcome measures and effect‐size calculation, the present findings suggest that STv may produce clinically meaningful reductions in voice‐related distress that warrant further investigation in controlled trials.

It is also noteworthy that these improvements were achieved using a relatively brief schema therapy intervention of approximately 20–30 sessions. Standard schema therapy for BPD is typically delivered over 18 months to 3 years, often involving weekly or twice‐weekly sessions and substantially greater treatment intensity (Giesen‐Bloo et al. 2006). The present intervention was also substantially briefer than the schema therapy context described by Baelemans et al. (2025) in BPD voice hearing individuals, whose participants had received treatment within a program lasting approximately 25 months. Although the studies are not directly comparable, this difference further highlights that STv was evaluated here as a focused, time‐limited adaptation for routine practice, and although preliminary, still with some promising effects. The abbreviated format used in the present study was developed pragmatically to reflect the realities of routine Australian psychological practice, where access to long‐term psychotherapy is often constrained by funding. Many clients attending specialist voice hearing services are unemployed or receiving disability or income‐support payments, making prolonged privately funded treatment financially unfeasible, while government‐funded psychology programs typically provide only time‐limited episodes of care. Dosage has been found to relate to outcome in CBT treatments with findings supporting that 25 sessions is better than 15 (Lincoln et al. 2016). The present findings therefore suggest that meaningful reductions in voice‐related distress can be achieved in short‐term frameworks. Whether larger number of schema sessions produce further improvement is an area for further research.

The third aim was to examine preliminary changes in the secondary clinical outcomes and the schema modes most directly targeted by STv. Improvements were observed across several secondary outcomes, with significant reductions in negative affect, emotion regulation difficulties and Punitive Parent mode. Although changes in voice severity, negative voice content and Vulnerable Child mode were of moderate effect size in the expected direction, these did not reach statistical significance, while no significant changes were observed in positive voice content or Healthy Adult mode (though again, these moved in the expected direction). Given the exploratory nature of these analyses and the relatively small sample, these findings should be interpreted cautiously. Nevertheless, the observed pattern of change provides preliminary support for the theoretical mechanisms underpinning STv.

Of particular interest was the significant reduction observed in Punitive Parent mode, as this was one of the principal mechanisms targeted by the intervention. Emerging theoretical work has proposed that many hostile and critical voices can be understood as manifestations of internalised Punitive Parent modes, while simultaneously activating the hearer's Vulnerable Child mode (Bartulovich et al. 2025; Newton et al. 2026). STv was specifically designed around this conceptualisation, aiming to reduce the influence of punitive voices and dysfunctional parent modes while strengthening the Healthy Adult's capacity to respond adaptively to the Vulnerable Child's unmet emotional needs. Although preliminary, the present findings provide some of the first treatment evidence consistent with this theoretical model.

The present findings also compare favourably with our earlier programme of research investigating ImRs for trauma‐affected voice hearers. In an initial case series and subsequent open trial, ImRs produced moderate‐to‐large reductions in voice‐related distress (*d* = 0.74) following a brief 10–20 session intervention (Paulik et al. 2019; Strachan, McEvoy, Arntz, et al. 2024). The larger effect observed in the present study (*d* = 1.18) may suggest that embedding ImRs within a broader schema therapy framework may enhance treatment effects by addressing additional maintaining mechanisms beyond trauma memories alone. One possible explanation is that, rather than focusing primarily on modifying trauma memories, STv also directly targets the punitive voice and the individual's ongoing relationship with their voice(s). STv used both chair‐work and ImRs as core experiential methods, with the relative emphasis determined by the individual formulation. The present service evaluation did not quantify exposure to each treatment component, so the independent contribution of ImRs, chair‐work or other schema therapy methods cannot be determined from these data.

Through chair‐work, ImRs and other experiential techniques, the therapy aims not only to reduce the influence of Punitive Parent modes but also to strengthen Healthy Adult functioning and responsiveness to the unmet needs of the Vulnerable Child mode and to put limits on the dysfunctional parent modes (Young et al. 2003). Such changes may enable individuals to relate differently to their voices, thereby reducing voice‐related distress even when voices themselves do not substantially diminish. This interpretation is consistent with the current pattern of findings, in which large reductions were observed in voice distress despite more modest changes in voice severity. However, these proposed mechanisms remain speculative and should be examined directly in future comparative and mediation studies. This pattern is consistent with the therapeutic goal of helping individuals change their relationship with their voices, rather than necessarily eliminating the voices themselves.

### 4.1 Clinical Implications

Although preliminary, the present findings suggest several potential implications for clinical practice. First, they support the value of extending assessment beyond voice phenomenology alone to include the underlying schema modes that may maintain voice‐related distress. Exploring whether voices resemble dysfunctional parent modes, the emotional states activated within the hearer (e.g., Vulnerable Child mode), and the individual's capacity to access Healthy Adult functioning may enhance case formulation and help guide intervention selection.

Taken together with emerging work linking voice phenomenology and content with schema modes (Bartulovich et al. 2025; Newton et al. 2026), the present findings suggest that schema therapy may offer a useful formulation framework for some help‐seeking voice hearers. Within that framework, experiential techniques such as ImRs and chair‐work may provide practical methods for modifying punitive internalised relationships and strengthening more adaptive responding, consistent with the qualitative observations of Baelemans et al. (2025). If replicated in controlled trials, the present findings suggest that a relatively brief, formulation‐driven adaptation of schema therapy may represent a feasible treatment option for trauma‐affected voice hearers within routine mental health services.

### 4.2 Limitations

These findings should be interpreted in light of several limitations. First, this was a naturalistic, uncontrolled service evaluation conducted within routine clinical practice. Although this design enhances ecological validity and provides information regarding the feasibility and potential utility of STv in a real‐world clinical setting, causal conclusions regarding treatment efficacy cannot be drawn. Improvements may reflect nonspecific therapeutic factors, spontaneous recovery, regression to the mean or concurrent interventions. Randomised controlled trials are therefore required to establish efficacy. Second, the sample size was relatively small, limiting statistical power, particularly for secondary outcomes and schema mode analyses. It is therefore possible that some clinically meaningful changes were not detected. Replication in larger samples is needed to confirm the robustness of the present findings. Third, outcomes were assessed only immediately following treatment. As schema therapy aims to produce enduring changes in maladaptive schemas, modes and interpersonal functioning, longer‐term follow‐up will be important to determine whether improvements are maintained over time. Conducting the study within routine clinical practice meant that treatment length, therapist allocation, medication regimens and changes (which were not experimentally controlled) and participant characteristics reflected the realities of service delivery rather than the tightly controlled conditions typical of efficacy trials. Although this introduces additional variability, it also enhances the clinical relevance and translational value of the findings. All participants were taking psychotropic medication, and medication regimens and changes were not experimentally controlled. Consequently, the contribution of medication to changes in voice‐related outcomes cannot be separated from treatment or other concurrent influences in this naturalistic evaluation. Finally, the sample was diagnostically heterogeneous, but it was not an entirely unselected transdiagnostic sample. Most participants had a psychotic disorder, BPD or both (though with additional co‐morbid diagnoses common), reflecting the clinical selection criteria for STv. The findings therefore may not generalise to voice hearers without substantial emotion‐regulation difficulties or trauma‐linked voice experiences.

## 5 Conclusion

STv represents a promising, mechanism‐focused approach for treating distressing voices by targeting Punitive Parent, Vulnerable Child and Healthy Adult modes. In this naturalistic service evaluation, STv was feasible to deliver within routine clinical practice and was associated with clinically meaningful reductions in voice‐related distress and improvements in key schema modes. Although the uncontrolled design precludes conclusions regarding efficacy, the findings provide preliminary support for the schema mode model of voice hearing and justify further evaluation of STv in larger controlled trials. Future controlled studies with larger samples and repeated assessment of schema modes are needed not only to establish efficacy but also to test whether changes in Punitive Parent, Vulnerable Child and Healthy Adult modes precede and help explain later reductions in voice‐related distress.

## Conflicts of Interest

The authors declare no conflicts of interest.

## Supporting information


**Data S1:** STv Intervention Structure.

## Data Availability

The data that support the findings of this study are available on request from the corresponding author. The data are not publicly available due to privacy or ethical restrictions.
