# Supplementary material for: Schema Therapy for Voice Hearers: A Case Series Study
Source: Clin Psychol Psychother. 2026 Oct 7;33(5):e70349. doi: 10.1002/cpp.70349 (PMC13646041; doi:10.1002/cpp.70349)
Supplement: Supplementary file 1 [file CPP-33-e70349-s001.docx]

**Supplementary Material 1. STv Intervention Structure**

STv sessions followed a consistent but flexible structure (the overall structure is shown in Table 2). Initial sessions of STv typically focus on assessment and exploration of participants’ developmental experiences, unmet emotional needs, early maladaptive schemas, and schema modes. A collaborative mode map is then developed, integrating participants’ voices into the formulation and identifying individualised treatment goals. The primary treatment phase focuses on reducing the influence of dysfunctional parent modes and associated critical, hostile, or demanding voices, predominantly through experiential interventions including chair-work and imagery rescripting. Chair-work is used to explore and alter relationships between schema modes and relevant associated voices. Imagery rescripting targets memories connected to the development and maintenance of dysfunctional modes and voices. Throughout treatment, there is a concurrent focus on strengthening the Healthy Adult mode and increasing its capacity to recognise and respond to vulnerable child needs. Maladaptive coping modes are also addressed when these maintain difficulties or interfere with experiential work, such as the instance where the detached protector coping mode prevents the client from being emotionally online in session and thus compromises the effectiveness of chair or imagery work. The relative emphasis and number of sessions devoted to each treatment component are formulation driven.

Sessions typically started with the client being asked to close their eyes and connect to their feelings and where they experience them in their body, and then to silently reflect on what they feel they need from themselves, from their therapist, and from others that day to help get their needs met. The participant’s current experiences and between-session work are then reviewed and understood with reference to the collaboratively developed mode map. Where appropriate, imagery is used to facilitate emotional engagement and identify activated modes and underlying needs, which then may become the focus of the experiential work that session. The majority of each session is devoted to experiential work targeting the mode or voice identified as most clinically relevant (predominantly those mapping on to the Dysfunction Parental Modes), supplemented by cognitive and behavioural techniques as indicated. Sessions conclude with reflection on the therapeutic work and agreement on relevant between-session tasks. Clients were given a transitional object of a diary early in therapy and were asked to make notes after each session and use the diary for personal reflections. Towards the end of treatment, the focus shifts towards consolidating the Healthy Adult mode, developing strategies for responding to dysfunctional modes and associated voices, and a schema relapse prevention (where for each of their top three schemas they identified common triggers for schema activation; which modes and voices are most commonly activated when that schema is triggerd; and ways the Healthy Adult can best soothe the child modes and get needs met).
